# Supplementary material for: Content-rich biological network constructed by mining PubMed abstracts
Source: BMC Bioinformatics. 2004 Oct 8;5:147. doi: 10.1186/1471-2105-5-147 (PMC528731; doi:10.1186/1471-2105-5-147)
Supplement: Additional File 2 — The original results of the above study (non-essential files are deleted to keep the file size under the limit set by BMC bioinformatics). [file 1471-2105-5-147-S2.bz2 › chilibotAdditionalFile2/dip10/433ID7622448E10378/html/CCL3_CCR3.html]

 


 **CCL3** and **CCR3** 
  
Found 21 abstracts in PubMed, retrieved 10.  
 

 What does Google say? 
 PDF only 
| .edu only 

---

**Interactive relationship** (e.g. stimulation, inhibition, etc)

**Neutral relationship**- We and others have shown that the human beta chemokine  **CCL3**  L1, unlike its related non allelic isoform  **CCL3** , has high affinity for the chemokine receptors D6,  **CCR3**  and CCR5.  Ref: 12355456 Eur J Immunol, 2002

**Non-interactive relationship** (e.g. studied together, co-existance, homology, etc.)

- Using self organizing maps, we identified nine genes CD4,  **CCR3** , CCR5, LT beta, MIP 1 alpha  [ **CCL3** ] , MIP 2, CD8 alpha, IP 10, and RANTES, each with a unique profile of transcriptional expression, that reproduce the differentiation of states of rejection in dendrograms.  Ref: 11818457 J Leukoc Biol, 2002
